# Deep Learning for Retail Product Recognition: Challenges and Techniques

**DOI:** 10.1155/2020/8875910

**Published:** 2020-11-12

**Authors:** Yuchen Wei, Son Tran, Shuxiang Xu, Byeong Kang, Matthew Springer

**Affiliations:** Discipline of ICT, School of TED, University of Tasmania, Launceston, Tasmania, Australia

## Abstract

Taking time to identify expected products and waiting for the checkout in a retail store are common scenes we all encounter in our daily lives. The realization of automatic product recognition has great significance for both economic and social progress because it is more reliable than manual operation and time-saving. Product recognition via images is a challenging task in the field of computer vision. It receives increasing consideration due to the great application prospect, such as automatic checkout, stock tracking, planogram compliance, and visually impaired assistance. In recent years, deep learning enjoys a flourishing evolution with tremendous achievements in image classification and object detection. This article aims to present a comprehensive literature review of recent research on deep learning-based retail product recognition. More specifically, this paper reviews the key challenges of deep learning for retail product recognition and discusses potential techniques that can be helpful for the research of the topic. Next, we provide the details of public datasets which could be used for deep learning. Finally, we conclude the current progress and point new perspectives to the research of related fields.

## 1. Introduction and Background

The intention of product recognition is to facilitate the management of retail products and improve consumers' shopping experience. At present, barcode [1] recognition is the most widely used technology not only in research but also in industries where automatic identification of commodities is used. By scanning barcode marks on each product package, the management of products can be easily facilitated. Normally, almost every item on the market has its corresponding barcode. However, due to the uncertainty of the printing position of the barcode, it often requires time to manually find the barcode and assist the machine in identifying the barcode at the checkout counter. Based on a survey from Digimarc [2], 45% customers complained that, sometimes, it was not convenient to use barcode scanning machines. RFID (radio frequency identification) [3] has been applied in business fields with the growth of computer technology to enhance the automation of product identification. This technology automatically transmits data and information using radio frequency signals. RFID tags are placed on each product. Each tag has its specific number corresponding to a specific product, and the product is identified by wireless signal communication. Unlike the barcode, RFID tag data are readable without the line-of-sight requirements of an optical scanner. Definitely, RFID has shortcomings. Identifying multiple products still has a high error rate due to radio waves being blocked or influencing each other. Also, RFID labels are expensive and difficult to recycle, resulting in higher sales costs and sustainability issues [4].

As retail is evolving at an accelerated rate, enterprises are increasingly focusing on how to use artificial intelligence technology to reshape the retail industry's ecology and integrate online and offline experiences [5]. Based on the study from Juniper Research, the global spending by retailers on AI services will increase over 300% from $3.6 billion in 2019 to $12 billion in 2023 [6]. That is to say, the new innovative retail in the future may be completely realized by artificial intelligence technology. Also, with the improvement of living standards, supermarket staff and customers are greeted with more than countless retail products. In this scenario, a massive amount of human labour and a large percentage of the workload were required for recognising products so as to conduct goods management [7]. Furthermore, with the help of various electronic devices for photographing, image digital resources of products are growing rapidly every day. As such, for a tremendous amount of image data, how to effectively analyze and process them, as well as to be able to identify and classify the products in supermarkets, has become a key research issue in the product recognition field. Product recognition refers to the use of technology which is mainly based on computer vision methods so that computers can replace the process of manually identifying and classifying products.

Implementing automatic product recognition in grocery stores through images has a significant impact on the retail industry. Firstly, it will benefit the planogram compliance of products on the shelf. For instance, product detection can identify which items are missing from the shelf to remind the store staff to replenish the products immediately. It is observed that when an optimized planogram is 100% matched, sales will be increased by 7.8% and profit by 8.1% [8]. Furthermore, image-based commodity identification can be applied to automatic self-checkout systems to optimize the user experience of checkout operations. Global self-checkout (SCO) shipments have steadily increased between 2014 and 2019. Growing numbers of SCOs have been installed to reduce retailers' costs and enhance customer experience [9, 10]. The research in [11, 12] demonstrates that customers' waiting time for checkout operations has a negative influence on their shopping satisfaction, which is to say that applying a computer-vision-based product recognition in SCOs benefits both retailers and customers. Thirdly, product recognition technology can assist people who are visually impaired to shop independently, which is conducive to their social connectivity [13]. Traditional shopping methods usually require assistance from a sighted person because it can be difficult for a person who is visually impaired to identify products by their visual features (e.g., price, brand, and due date), making purchase decisions difficult [14].

In general, retail product recognition problems can be described as an arduous instance related to image classification [15, 16] and object detection problems [17–19]. During the last decade, deep learning, especially in the domain of computer vision, has achieved tremendous success and has become the core solution for image classification and object detection. The primary difference between deep learning and traditional pattern recognition methods is that the former can directly learn features from image data rather than using manually designed features. Another reason for the strong ability of deep learning is the deeper layers that can extract more precise features than traditional neural networks. The above advantages enable deep learning methods to bring new ideas to solve some important computer vision problems such as image segmentation and keypoint detection. Recently, a few attempts have been applied to the retail industry, following with state-of-the-art results [20–22]. In the meanwhile, some automated retail stores have emerged, such as Amazon Go (https://www.amazon.com/b?ie=UTF8&node=16008589011) and Walmart's Intelligent Retail Lab (https://www.intelligentretaillab.com/), which indicate that there is interest in unmanned retail with deep learning.

Deep learning-based retail product recognition has increasingly attracted researchers, and plenty of work has been done in this field. However, it appears that there are very few reviews or surveys that summarize existing achievements and current progress. We collected over a hundred related publications through Google Scholar, IEEE Xplore, and Web of Science, as well as some great conferences such as CVPR, ICCV, IJCAI, NIPS, and AAAI. As a result, only two formally published surveys [4, 23] came to light, which studied the detection of products on the shelf in retail stores. The scenario of recognising products for self-checkout systems has been neglected in their surveys, which is also a complex task that needs to be solved for the retail industry.

In the published article [23], authors reviewed 24 papers and proposed a classification of product recognition systems. Nevertheless, deep learning methods are not mentioned in this paper. Another related survey was from [4], and the authors presented a brief study on computer vision-based product recognition in shelf images. However, this survey does not focus on the field of deep learning: most of the methods presented are based on hand-crafted features. Therefore, with the rising popularity and potential applications of deep learning in retail product recognition, a new comprehensive survey is demanded for a better understanding of this research field.

In this paper, we present an extensive literature review of current studies on deep learning-based retail product recognition. Our detailed survey presents challenges, techniques, and open datasets for deep learning-based product recognition. It offers meaningful insights into advances in deep learning for retail product identification. It also serves as a guideline for researchers and engineers who have just started researching the issue of product recognition, with the purpose that they will find the problems that need to be studied quickly. In summary, there are three points for the contribution of this paper: (1) for the implementation of deep learning methods in product identification, we provide a comprehensive literature review. (2) We propose current problem-solving techniques according to the complexity of retail product recognition. (3) We discuss the challenges and available resources and identify future research directions.

The rest of this article will be structured as follows: Section 2 introduces the overview of computer vision methods for product recognition.  Section 3 presents the challenges in the field of detecting grocery products in retail stores.  Section 4 gives current techniques to solve the complex problems.  Section 5 describes the publicly available datasets and analyzes their particular application scenarios. Finally, Section 6 draws the conclusion and provides directions for future studies.

## 2. Computer Vision Methods in Retail Product Recognition

### 2.1. Classic Methods

**Figure 1:**
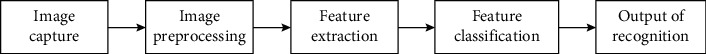
The flowchart of the product image recognition system.

With computer vision's rapid growth, researchers have been drawn to product recognition using the technology. Product recognition is realized by extracting features on the image of the package. The composition of the product image recognition system is shown in Figure 1. (1) Image capture: collecting images from cameras and mobile phones. (2) Image preprocessing: reducing noise and removing redundant information to provide high-quality images for subsequent operations. It mainly includes image segmentation, transformation, and enhancement. (3) Feature extraction: the analysis and processing of image data to determine the invariant characteristics in the image. (4) Feature classification: after a certain image feature is mapped to the feature vector or space, a specific decision rule is applied to classify the low-dimensional feature to make the recognition result accurate. (5) The output of recognition: the pretrained classifier is employed to predict the category of the retail product.

The core of product recognition is whether accurate features can be extracted or not. SIFT [24, 25] and SURF [26, 27] are the best representatives of traditional feature extraction technology. In 1999, Lowe suggested SIFT, paying greater attention to local information, where an image pyramid was established to solve the problem of multiscale features. SIFT features have many advantages, such as rotation invariance, translation invariance, and scale infinity, which are the most widely used hand-crafted features before deep learning. In 2006, based on the foundation of SIFT, some researchers proposed SURF features to improve calculation speed. SIFT has been used as a feature extractor for product classification in [13], and the SURF algorithm has been applied in [28] to detect the out-of-stock and misplaced products on shelves. However, due to the features extracted by SIFT and SURF being hand-crafted, it is unable to reflect all sufficient information fully. Thus, researchers are increasingly interested in deep learning for end-to-end training to extract effective features.

### 2.2. Deep Learning

Deep learning is often regarded as a subfield of machine learning. The vital objective of deep learning is to learn deep representation, i.e., to learn multilevel representation and abstraction from information [29]. Initially, the concept of deep learning (also known as deep structured learning) was proposed by authoritative scholars in the field of machine learning in 2006 [30]. After a short while in 2006, Hinton and Salakhutdinov presented the methods of unsupervised pretraining and fine-tuning to solve the vanishing gradient problem [31]. After that year, deep learning became a research hotspot. In 2007, a greedy layer-wise training strategy was provided to optimize the initial weights for deep networks [32]. ReLU (rectified linear unit) was defined in 2011 to preserve more information among multiple layers which could restrain the vanishing gradient problem [33]. The dropout algorithm [34] was proposed in 2012 to prevent overfitting, and it helped improve the deep network performance.

In the field of computer vision, deep neural networks have been exploited with the improvement of computing power from computer hardware, particularly thanks to the implementation of GPUs in image processing. Nowadays, the application of deep learning in retail product recognition primarily covers the following two elements: (1) image classification: this is a fundamental task in computer vision, which seeks to divide different images into different categories. The performance of classifying images with computers is already better than humans. (2) Object detection: it refers to detecting objects with rectangular boxes while categorising images. In the last few years, with the ongoing growth of deep learning, many scientists and developers have built and optimized some deep learning frameworks to help speed-up training and forecast procedures, such as Caffe [35], TensorFlow [36], MXNet [37], and PyTorch [38], which are the most common frameworks that make the use of deep learning methods much easier for scientists.

#### 2.2.1. Convolutional Neural Networks

The success of deep learning in computer vision profits from convolutional neural networks (CNNs), which are inspired by the biology research of the cat's visual cortex [39]. LeCun et al. first proposed to employ convolutional neural networks to classify images in 1988 [40]. They conceived the LeNet convolutional neural network model that had seven layers. After training on a dataset which contained 32*∗*32 handwritten characters, this model had been successfully applied to the digital identification of checks. Opportunely, the structure of the CNN and training techniques have been experiencing strong advances since 2010, benefiting from the ImageNet Large-Scale Visual Recognition Challenge. Also, with the advance of computing power from GPUs, deep learning has undoubtedly become a phenomenon. After the year of 2010, a series of network structures such as AlexNet [21], GoogLeNet [41], VGG [42], and ResNet [43] were devised for image classification based on LeNet [40]. Recently, the CNN becomes able to classify 3D objects, which is named as a multiview CNN [44]. The multiview CNN has shown a remarkable performance on image classification tasks by inputting multiple images to the networks [45]. In the age of big data, it enables researchers to select large datasets to train complex structures of networks that output more accurate results. In conclusion, big data and deeper networks are the two key elements for the success of deep learning, and these two aspects accelerate each other.

#### 2.2.2. Deep Learning for Object Detection

CNNs have been the major deep learning technique for object detection. Therefore, all the deep learning models discussed in this paper are based on the CNN. In order to detect various objects, it is essential to conduct region extraction on different objects before image classification. Before deep learning, the common regional extraction method is the sliding window algorithm [46]. This algorithm is a traditional method, identifying the object in each window by sliding the image. The sliding window strategy is inefficient, which requires a very large amount of calculation. After incorporating deep learning into this field, the object detection techniques can be classified into two categories: the two-stage model (region proposal-based) and the one-stage model (regression/classification-based) [47]. The two-stage model requires a region proposal algorithm to find out the possible location of the object in a graph. It takes advantage of textures, edges, and colours from the image to ensure a high recall rate, while fewer windows (thousands or even hundreds) are selected. In the R-CNN algorithm [48], an unsupervised region proposal method, selective search [49], is introduced, combining the power of both exhaustive search and segmentation. Although this method has improved computing speed, it still needs to implement a CNN calculation for every region proposal. Then, Fast R-CNN [18] was developed to reduce the repeated CNN calculation. Ren et al. proposed a region proposal network (RPN) [50] by using a deep network while sharing features with the classification network. The shared features not only avoid the time consumption caused by recalculation but also improve the accuracy. The Faster R-CNN algorithm, based on the RPN, is presently the mainstream technique of object identification, but it does not satisfy the computing speed criteria in real time. Compared with the two-stage method, the one-stage method computes faster because it skips the region proposal stage, and then objects' locations and categories are directly regressed from multiple positions of the image. YOLO [51] and SSD [52] are the most representative algorithms, greatly speeding up detection, while accuracy is inferior to the two-stage method.

#### 2.2.3. Product Recognition Based on Deep Learning

**Figure 2:**
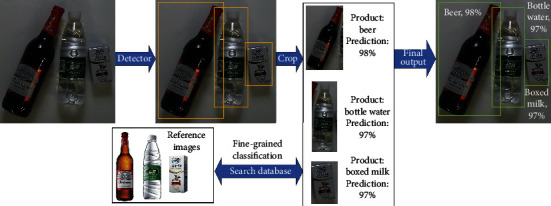
A typical pipeline of image-based product recognition.

Deep learning has made a research on object detection to develop rapidly. In this work, we perceive product recognition as a particular research issue related to object detection. At present, computer vision has achieved widespread use already; however, its application of product image recognition is still less perfect. A typical pipeline of image-based product recognition is shown in Figure 2, and the product images are from the RPC dataset [7]. In general, as regional proposals, an object detector was used to acquire a set of bounding boxes. Then, several single-product images are cropped from the original image, which contains multiple products. Finally, each cropped image can be input into the classifier, making the recognition of the products an image classification task.

In the last few years, some large technology companies have applied deep learning methods for recognising retail products in order to set up unmanned stores. Amazon Go (https://www.amazon.com/b?ie=UTF8&node=16008589011) was the first unmanned retail store that was open to the general public in 2018. There are dozens of CCTV cameras in the Amazon Go store, and by using deep learning methods, the cameras are able to detect the customers' behaviour and identify the products they are buying. Nevertheless, the recognition accuracy with the images still leaves much to be desired. Hence, some other technologies, including Bluetooth and weight sensors, are also employed to ensure the retail products can be identified correctly. Shortly after the Amazon Go store, a new retail store called Intelligent Retail Lab (IRL) (https://www.intelligentretaillab.com/) was designed by Walmart in 2019 to inspect the application of artificial intelligence in retail services. In IRL, deep learning was exploited with cameras to automatically detect the out-of-stock products and alert staff members when to restock. Furthermore, a number of intelligent retail facilities, such as automatic vending machines and self-serve scales, have emerged recently. A Chinese company, DeepBlue Technology (https://en.deepblueai.com/), has developed automatic vending machines and self-checkout counters based on deep learning algorithms, which can accurately recognize commodities by using the cameras. Malong Technologies (https://www.malong.com/en/home.html) is another well-known business in China that aims to provide deep learning solutions for the retail industry. The facilities from Malong Technologies include AI Cabinets that perform automatic product recognition using the computer vision technology and AI Fresh that enables identification of fresh products on a self-serve scale automatically. However, all the deep learning-based facilities are still in their early stages and have not entered the widespread implementation. More researches and practical tests need to be done in this area.

Based on the above review of current studies, we suggest that deep learning is an advanced method, as well as a growing technique, for retail product recognition; however, more research is needed in this area.

## 3. Challenges

As mentioned in the Introduction section, the peculiarity of retail product recognition makes it more complicated than common object detection since there are some specific situations to consider. In this section, we generalize the challenges regarding retail product recognition and classify them into the four aspects shown in the following.

### 3.1. Large-Scale Classification

The number of distinct products to be identified in a supermarket can be enormous, approximately several thousands, for a medium-sized grocery store that far exceeds the ordinary capability of object detectors [53].

**Figure 3:**
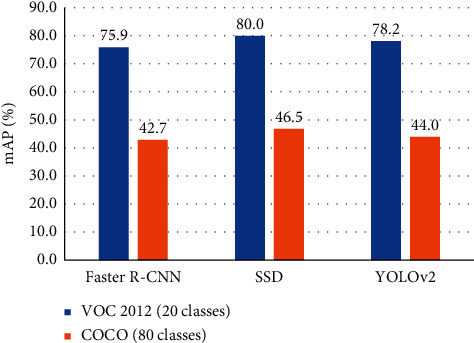
Comparative results on VOC 2012 and COCO test sets.

Currently, YOLO [17, 51, 54], SSD [52], Faster R-CNN [50], and Mask R-CNN [55] are state-of-the-art object detection methods, which evaluate their algorithms with PASCAL VOC [56] and MS COCO [57] datasets. However, PASCAL VOC only contains 20 classes of objects, and MS COCO contains photos of 80 object categories. This is to say that the current object detectors are not appropriate to apply to retail product recognition directly due to their limitations with large-scaled categories.  Figure 3 compares the results on VOC 2012 (20 object categories) and COCO (80 object categories) test sets with different algorithms, including Faster R-CNN, SSD, and YOLOv2. We only list three approaches of object identification to demonstrate that the precision of all detectors reduces dramatically when the number of classes rises. More comparative results can be found in [47].

Additionally, the data distribution of the VOC dataset is more than 70 percent of the images contain objects belonging to one category, and more than 50 percent involve only one instance per image. On average, each picture contains 1.4 categories and 2.3 instances. With regard to the COCO dataset, it contains an average of 3.5 categories and 7.7 instances per image. In a practical scenario of a grocery store, customers usually buy dozens of items from more than ten categories. Therefore, based on the data above, it illustrates that the recognition of the retail product has its peculiarities compared with common object detection. As a result, how to settle this practical problem is still an open question.

### 3.2. Data Limitation

Deep learning-based approaches require a large amount of annotated data for training, raising a remarkable challenge in circumstances where only a small number of examples are available [21]. In Table 1, it lists some open-source tools that can be used for image labelling. These tools have been divided into two categories: bounding box and mask. The bounding box category includes tools that can label the object with a bounding box, while tools in the mask category can be useful for image segmentation. These image captioning tools require manual labour to label every object in each image. Normally, there are at least tens of thousands of training images in a general object detection dataset, apparently indicating that creating a dataset with enough training data for deep learning is time-consuming work.

**Figure 4:**
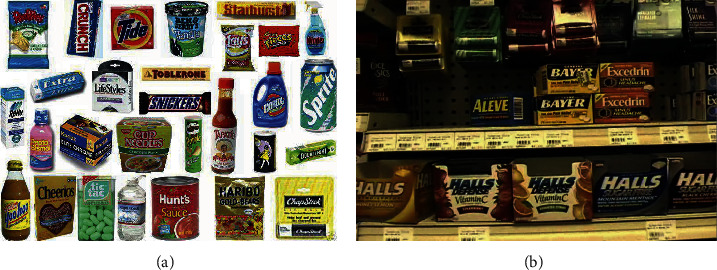
GroZi-120: samples of training images (a) and testing images (b).

Furthermore, with regard to grocery product recognition in retail scenarios, the majority of the training data is acquired in ideal conditions instead of practical environments [58]. As a sample shown in Figure 4, training images are usually taken with the same single product from several different angles in a rotating platform, while testing images are from real conditions, which contain multiple products per image with a complex background.

**Figure 5:**
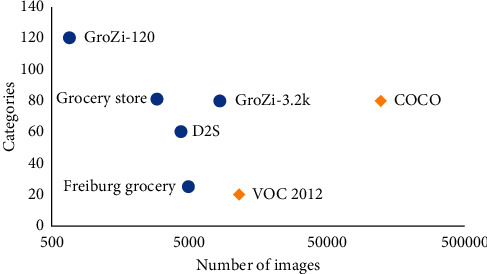
Comparison between common object datasets and retail product datasets.

Last but not least, the majority of scholars aims to perfect the dataset of common object detection, such as VOC 2012 and COCO, which results in the data limitation issue to product recognition.  Figure 5 illustrates that compared with common object datasets, retail product datasets have fewer images with more classes. Therefore, it is necessary to provide a larger dataset for training a deep learning model when we want that model to be able to recognize objects from various categories.

Based on the above realization, we can conclude that the data shortage is a real challenge to retail product recognition.

### 3.3. Intraclass Variation

Intraclass classification, also known as subcategory recognition, is a popular research topic both in the industrial and academic areas, aiming at distinguishing subordinate-level categories. Generally, identifying intraclass objects is a very challenging task due to the following: (1) objects from similar subordinate categories often have only minor differences in a certain area of their appearance. Sometimes, this task is even difficult for humans to classify. (2) Intraclass objects may present multiple appearance variations with different scales or from various viewpoints. (3) Different environmental factors, such as lighting, backgrounds, and occlusions, may have a great impact on the identification of intraclass objects [59]. To solve this challenging problem, fine-grained object classification is required to identify subcategory object classes, which includes finding the subtle differences among visually similar subcategories. At present, fine-grained object classification is mainly applied to distinguish different species of birds [60], dogs [61], flowers [62], or different brands of cars [63]. Moreover, compared with datasets for common object classification, it is more difficult to acquire fine-grained image datasets, which require relevant professional knowledge to complete image annotations.

**Figure 6:**
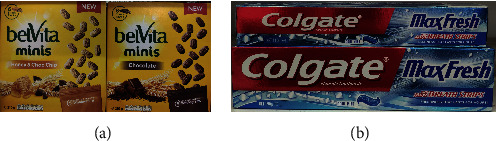
Intraclass products with different flavours (a) (honey flavour and chocolate flavour) and size (b) (110 g and 190 g).

Due to the visual similarity in terms of shape, colour, text, and metric size between intraclass products, retail products are really hard to be identified [64]. It can be difficult for customers to determine the difference between two flavours of cookies of the same brand; we can expect it to be complex for computers to classify these intraclass products.  Figure 6(a) demonstrates two products with different flavours only have minute differences of colour and text on the package.  Figure 6(b) shows the visually similar products with different sizes. Additionally, up to now, there have been no specific fine-grained datasets for retail product recognition. The fine-grained classification methods usually require additional manual labelling information. Without enough annotation data, it is more demanding to use deep learning methods to identify similar products.

### 3.4. Flexibility

In general, with the increasing number of new products every day, grocery stores need to import new items regularly to attract customers. Moreover, the appearances of existing products change frequently over time. Due to the reasons above, a practical recognition system should be flexible with no or minimal retraining whenever a new product/package is introduced [20]. However, convolutional neural networks always suffer from “catastrophic forgetting”—they are unable to recognize some previously learned objects when adapted to a new task [65].

**Figure 7:**
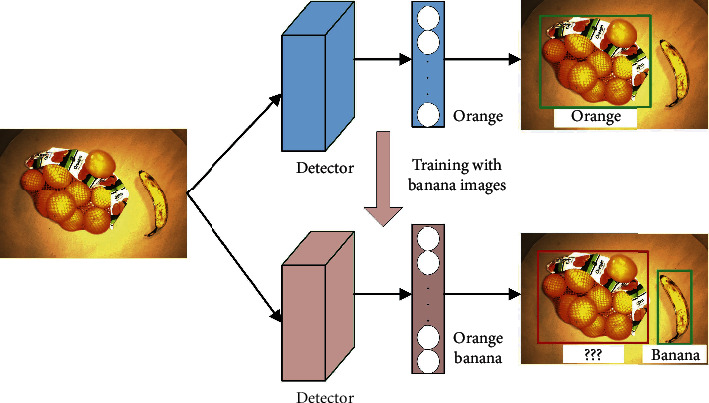
An example of introducing a new class to an existing retail product detector.

Figure 7 illustrates that, after training a detector with a new class, banana, it may probably forget the previous objects. The top detector is trained with a dataset including orange, so it can detect orange in the image. Then, introducing a new class, banana, to the detector, we train it only with banana images rather than with all the classes jointly. Finally, the bottom detector is generated, which can recognize the new class, banana, in the image. Nevertheless, this bottom detector fails to localize orange because of forgetting the original knowledge of orange.

Currently, top-performing image classification and object detection models have to be retrained completely when introducing a new category. It poses a key issue as collecting new training data and retraining networks can be time-consuming. Therefore, how to develop an object detector with long-term memory is a problem worthy of study.

## 4. Techniques

**Figure 8:**
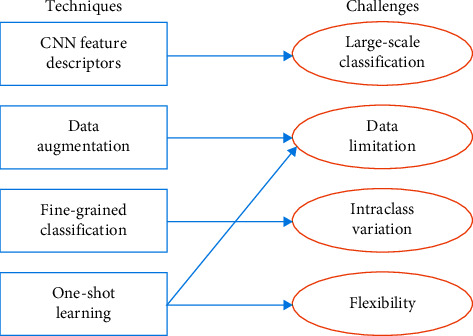
Techniques for challenges.

Concerning the four challenges proposed in Section 3, we refer to a considerable amount of literature and summarize current techniques related to deep learning, aiming to provide some references with which readers can quickly gain entrance to the field of deep learning-based product recognition. In this paper, we not only introduce the approaches in the scope of deep learning but also present some related methods that can be combined with deep learning to advance the recognition performance.  Figure 8 demonstrates the techniques' target for the proposed challenges.

### 4.1. CNN-Based Feature Descriptors

The key issue of image classification lies in the extraction of image features; by using the extracted features, the images can be categorized into different classes. For the challenge of large-scale classification in Section 3, the traditional hand-crafted feature extraction methods, e.g., SIFT [24, 25] and SURF [26, 27], seem to be overtaken by the convolutional neural network (CNN) [66] due to their limitations for exploring deep information from images. At the moment, CNN is a promising technique that has a strong ability to create embedding for different classes of objects. Some researchers have attempted to use the CNN for feature extraction [48, 67–69].  Table 2 shows the related works with CNN-based feature descriptors for retail product recognition.

In [72], Inception V3 [81] has been used to implement image classification of eight different kinds of products on the shelves. The drawback is that the prediction accuracy of the images from real stores only reaches 87.5%, and that needs to be improved. Geng et al. [74] employed VGG-16 as the feature descriptor to recognize the product instances, achieving recognition for 857 classes of food products. In this work, VGG-16 is integrated with recurring features and attention maps to improve the performance of grocery product recognition in the real-world application scenario. The authors also implemented their method with ResNet; then, 102 grocery products from CAPG-GP (the dataset built in this paper) were successfully classified with the mAP of 0.75. Another notable work using ResNet is from [22] that introduces a scale-aware network for generating product proposals in supermarket images. Although this method does not aim to predict the product categories, it can accurately perform the object proposal detection for the products with different scale ratios in one image, which is a practical issue in supermarket scenarios. In [71], authors considered three different popular CNN models, VGG-16 [42], ResNet [43], and Inception [70], in their approach and performed the K-NN similarity search extensively with the output of the three models. Their method was evaluated with three grocery product datasets, and the largest one contained 938 classes of food items. AlexNet was exploited in [53] to compute visual features of products, combining deep class embedding into a CRF (conditional random field) [82] formulation, which enables classifying products with a huge number of classes. The benchmark in this paper involved 24,024 images and 460,121 objects, and each object belonged to one of 972 different classes. The above method can only be applied to a small retail store as all of them recognize up to 1,000 classes of products, while a stronger ability to classify more categories of items is required for medium-sized and large-sized retail stores.

Recent works have tried to realize large-scale classification, e.g., Tonioni et al. and Karlinsky et al. [20, 21] proposed approaches that can detect several thousand product classes. In [20], the backbone network for its feature descriptor is VGG, from which a global image embedding is obtained by computing MAC (maximum activations of convolutions) features [83]. This research is dealing with the products belonging to 3,288 different classes of food products. Finally, Tonioni et al. obtained state-of-the-art results of precision and recall, as 57.07% PR and 36.02% mAP, respectively. In the work of Karlinsky et al. [21], the CNN feature descriptor is based on fine-tuning a variant of the VGG-F network [84], which deploys the first 2–15 layers of VGG-F trained on ImageNet [85] unchanged. As a result, the authors presented a method to recognize each product category out of a total of 3,235, with an mAP of 52.16%. According to the data from these two papers, it is obvious that the recognition accuracy, including precision and recall, still has a considerable space for improvement to implement this technique in the retail industry area.

Lately, the most popular object detector YOLO9000 [54] has proposed a method that can detect 9,000 object classes by using revised Darknet [86]. Unfortunately, YOLO9000 has been trained with millions of images, which is infeasible in the case of training a product recognition model due to the high annotation costs. However, the success of YOLO9000 illustrates the potential ability of the CNN to achieve a large-scale level of classification (thousands of classes). As for the problem of how to produce more data available for training, we will discuss in the next section.

### 4.2. Data Augmentation

It is common knowledge that deep learning methods require a large number of training examples; nevertheless, acquiring large sets of training examples is often tricky and expensive [87]. Data augmentation is a common technique used in deep network training to handle the shortage of training data [78]. This technique uses a small number of images to generate new synthetic images, aiming to artificially enlarge the small datasets to reduce the overfitting [15, 88]. In this paper, we define the current mainstream approaches into two categories: common synthetic methods and generative models. The existing publications are listed in Table 3.

#### 4.2.1. Common Synthesis Methods

The common methods for image data augmentation generate new images through translations, rotations, mirror reflections, scaling, and adding random noise [15, 90, 91]. A significant attempt can be found in the work of Merler et al. [89]; synthetic samples were created from images under ideal imaging conditions (referred to as in vitro) by applying randomly generated perspective distortions.

**Figure 9:**
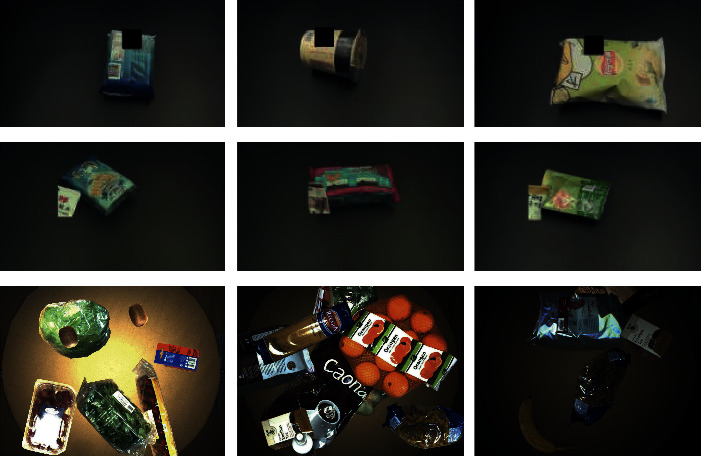
First two rows show examples of occlusion simulation in [79], and the third row demonstrates example images from [80] under three different lightings.

The occlusion for each product is also a common phenomenon in real practice. In [22], the authors proposed a virtual supermarket dataset to let models learn in the virtual environment. In this dataset, the occlusion threshold is set to 0.9, which means the product occluded under the threshold 0.9 will not be labelled as the ground truth. UnrealCV [92] was employed to extract the ground truth of object masks from real-world images. Then, they manipulated the extracted object masks on a background of shelves and rendered 5,000 high-quality synthetic images. In this paper, some other aspects such as realism, the randomness of placement, products' overlapping, object scales, lighting, and materials were taken into account when constructing the synthetic dataset. By using the virtual supermarket dataset, they achieved identification of items in the real-world datasets without fine-tuning. Recently, Yi et al. [79] tried to simulate the situation of occlusion by overwriting a random region in the original image either by a black block or a random patch from another product. Then, they fine-tuned their Faster R-CNN detection model with in vitro (in ideal conditions) and in situ (in natural environments) data and obtained a relatively high rate in mAP and recall. In situ is divided into conveyor and shelf scenarios where the authors obtained the mAP of 0.84 on the conveyor and 0.79 on the shelf, respectively. Some synthetic samples are shown in the first two rows of Figure 9. Inadequately, the comparative experiments between the proposed algorithm and the other state-of-the-art algorithms are absent in this paper.

The work in [80] synthesizes new images containing multiple objects by combining and reorganizing atom object masks. Ten thousand new images were assembled, which contained one to fifteen objects randomly. For each generated image, the lighting, the class of object instances, the orientation, and the location in the image are randomly sampled. The last row in Figure 9 shows example synthetic images under three different lightings. By adding the 10,000 generated images to the training set, the AP on the test set has been improved to 79.9% and 72.5% for Mask R-CNN [55] and FCIS [93], respectively. By contrast, the achievement of AP is only 49.5% and 45.6% without the generated data.

To realize product recognition with a single example, researchers in [21] generated large numbers of training images using geometric and photometric transformations based on a few available training examples. In order to facilitate image augmentations for computer vision tasks, albumentations are presented in [94] as a publicly available tool that enables varieties of image transformation operations. Recent work in [95] has applied albumentations with a small training dataset and then trained the product detection model with the augmented dataset. The outcomes show that the model can attain reasonable detection accuracy with fewer images.

However, the common methods for generating new images have their limitations to simulate various conditions in the real world. Generative models are provided to prevent the models from learning various conditions illogically.

#### 4.2.2. Generative Models

Nowadays, generative models include variational autoencoder (VAE) [96] and generative adversarial networks (GANs) [97], gaining more and more attention due to the potential ability to synthesize in vitro images similar to those in realistic scenes for data augmentation [78]. Normally, generative models enrich the training dataset in two ways. One is generating new images with an object that looks similar to the real data. The synthetic images can directly increase the number of training images for each category. Another approach is the image-to-image translation, which is described as the issue of translating the picture style from the source domain to the target domain [98]. For example, if the target domain is defined as a practical scene in a retail store, this image transfer approach can improve training images to be more realistic, such as different lightings, views, and backgrounds. In Table 4, we list some state-of-the-art models that are based on the architectures of VAE and GAN for image generation and translation, respectively. The works displayed in the table prove that the models based on the GAN are powerful for producing new images as well as to enable the image-to-image transfer. Unfortunately, the approaches based on the strength of VAE have been unable to achieve image translation tasks up to now. The detailed research and application status of image synthesis with VAE and GAN are introduced in the following.

VAE has not been applied as an image creator in the domain of product recognition so far. The general framework of VAE comprises an “encoder network” and a “decoder network.” After training the model, we can use the “decoder network” to generate realistic images. In this paper, we present some successful cases of VAE in other classification and detection fields for reference. In [101], a novel layered foreground-background generative model trained in an end-to-end deep neural network using VAE is provided for generating realistic samples from visual attributes. This model was evaluated with the Wild (LFW) dataset [111] and the Caltech-UCSD Birds-200-2011 (CUB) dataset [60] which contained natural images of faces and birds, respectively. The authors have trained an attribute regressor to compare the differences between generated images and real data. Finally, their model achieved 16.71 mean squared error (MSE) and 0.9057 cosine similarity on the generated samples. Another noteworthy work is from [112], where the authors used a conditional VAE to generate the samples from the given attributes for addressing zero-shot learning problems. They tested this method on four benchmark datasets, AwA [113], CUB [60], SUN [114], and ImageNet [85], and gained state-of-the-art results, particularly in a more realistic generalized setting. These successful application examples of VAE manifest that VAE is a promising technique for data augmentation. With the increasing attention for product recognition, VAE will be applied in this field soon.

GAN, which was proposed in 2014, has been achieving remarkable efficiency in various research fields. The framework of the GAN consists of two models: a generator that produces fake images and a discriminator that estimates the probability that a sample is a real image rather than a fake one [97]. As a result, compared with common synthetic methods, the generator can be used to generate images that look more realistic.

With the advantage of generating realistic images, scholars have demonstrated the great potential of using the GAN and its variant [104–107] to produce images for enlarging the training set. For example, in [115], authors built a framework with structure-aware image-to-image translation networks, which could generate large-scale trainable data. After training with the synthetic dataset, the proposed detector provided a significant performance on night-time vehicle detection. In another work [116], a novel deep semantic hashing was presented, which combined with the semisupervised GAN, to produce highly compelling data with intrinsic invariance and global coherence. This method achieved state-of-the-art results with CIFAR-10 [117] and NUS-WIDE [118] datasets. A new image density model based on the PixelCNN architecture was established in [119], which could be used to generate images from diverse classes by simply conditioning on a one-hot encoding of that class. Zheng et al. employed the DCGAN to produce unlabeled images in [120] and then applied these new images to train the model for recognising fine-grained birds. This method has attained an enhancement of +0.6% over a powerful baseline [121]. In [122], CycleGAN was used to create 200,000 license plate images from 9,000 real pictures. Its result demonstrated an increase of 7.5 percentage points of recognition precision over a strong benchmark that was trained only with real data. The evidence above indicates that GANs are powerful tools for generating realistic images that can be used for training deep neural networks. It is likely that, in the near future, the experience of the above methods can be borrowed for improving the effects of product recognition.

**Figure 10:**
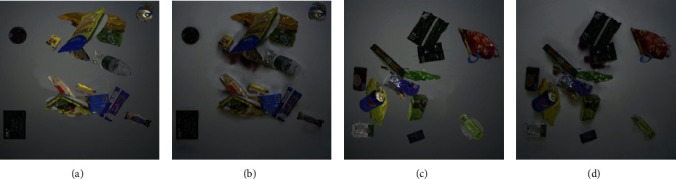
Synthesized checkout images (left) and the corresponding images generated by CycleGAN (right) from [7].

Although GANs have shown compelling results in the domains of general object classification and detection, there are very few works using GANs for product recognition. To the best of our knowledge, there are only three papers [7, 71, 78] attempting to exploit GANs to create new images in the field of product recognition. In the work of [7], the authors proposed a large-scale checkout dataset containing synthetic training images generated by CycleGAN [106]. Technically, they firstly synthesized images with object instances on a prepared background image. Then, CycleGAN was employed to translate these images into the checkout image domain. By training with the combination of translated images and original images, their product detector, feature pyramid network (FPN) [123], attained 56.68% accuracy and 96.57% mAP on average.  Figure 10 indicates the CycleGAN translating effects. Based on the work of Wei et al. [7], Li et al. [78] conducted further research through selecting reliable checkout images with the proposed data priming network (DPNet). Their method achieved 80.51% checkout accuracy and 97.91% mAP. In [71], GAN was deployed to produce realistic samples, as well as to play an adversarial game against the encoder network. However, the translated images in both [7, 78] only contain a simple background of flat colour. Considering the complex backgrounds of the real checkout counter and the goods shelf, how to generate retail product images in a more true-to-life setting is worthy of research.

### 4.3. Fine-Grained Classification

Fine-grained classification is a challenging problem in computer vision, which can enable computers to recognize the objects of subclass categories [124, 125]. Recently, a number of researchers and engineers have focused on the technique of fine-grained classification and already applied it in a significant number of domains with remarkable achievements, e.g., animal breeds or species [126–131], plant species [62, 131–133], and artificial entities [129, 130, 134–136]. Fine-grained retail product recognition is a more challenging task than general object recognition due to intraclass variance and interclass similarity. Considering the specific complications in product recognition in terms of blur, lighting, deformation, orientation, and the alignment of products in shelves, we summarized the existing product fine-grained classification methods into two categories, i.e., fine feature representation and context awareness.

#### 4.3.1. Fine Feature Representation

Fine feature representation refers to extracting fine features in a local part of the image to find the discriminative information between visually similar products. As a consequence, how to effectively detect foreground objects and find important local information has become a principal problem for fine-grained feature representation. According to the supervisory information for training the models, the fine feature representation methods can be divided into two categories: “strongly supervised fine feature representation” and “weakly supervised fine feature representation.”

*(1) Fine Feature Representation from Strongly Supervised Models*. The strongly supervised methods require additional manual labelling information such as a bounding box and part annotation. As mentioned in Section 3, the practical applicability of such methods has been largely limited by the high acquisition cost of annotation information. The classical methods include part-based R-CNN [127] and pose-normalized CNN [137].

In [127], part-based R-CNN is established to identify fine-grained species of birds. This method uses R-CNN to extract features from the whole-objects (birds) and local areas (head, body, etc.). Then, for each region proposal, it computes scores with features from an object and each of its parts. Finally, through considering synthetically with the scores of fine-grained features, this method achieves state-of-the-art results on the widely used fine-grained benchmark Caltech-UCSD bird dataset [60].

Branson et al. presented pose-normalized CNN in [137], and the fine-grained feature extraction process in this paper is as follows: (1) the DPM algorithm is used to detect the object location and its local areas. (2) The image is cropped according to the bounding boxes, and features are extracted from each cropped image. (3) Based on different parts, convolution features are extracted from multiple layers of the CNN. (4) These features are imported into one-vs-all linear SVMs (support vector machines) [138] to learn weights. Eventually, the classification accuracy of their method reached 75.7% on the Caltech-UCSD bird dataset.

In the domain of retail product recognition, the work in [139] can be considered as a solution for the fine-grained classification to some extent. The researchers designed an algorithm called DiffNet that could detect different products between a pair of similar images. They have labelled different products in each pair of images, and there is no need to annotate the constant objects. The consequence of this was that this algorithm achieved a relatively desirable detection accuracy of 95.56% mAP. The DiffNet would probably benefit the progress of product recognition, particularly for detecting the changes of the on-shelf products.

*(2) Fine Feature Representation from Weakly Supervised Models*. The weakly supervised techniques prevent the use of costly annotations such as bounding boxes and part information. Similar to the strongly supervised classification methods, the weakly supervised methods also require global and local features for the fine-grained classification. Consequently, the principal task of a weakly supervised model is how to detect the parts of the object and extract fine-grained features.

The two-level attention [126] algorithm is the first attempt to perform fine-grained image classification without relying on part annotation information. This method is based on a simple intuition: extracting the features from the object level and then focusing on the most discriminative parts that can be used for the fine-grained classification. The constellation [140] algorithm was proposed by Simon and Rodner in 2015. It exploits the features from the convolution neural network to generate some neural activation patterns that can be used to extract features from parts of the object. Another remarkable work is from [141], where the authors proposed novel bilinear models that contain two CNNs, A and B. The function of CNN A is to complete the localization of the object and its parts, while B is able to extract features of region proposals from CNN B. These two networks coordinate with each other and obtain 84.1% accuracy in the Caltech-UCSD bird dataset.

Regarding the fine-grained classification of retail products, some academic staff are beginning to take advantage of fine feature representation to identify subclass products. In [21], a CNN was proposed for improving the fine-grained classification performance, combined with scored short-lists of possible classifications from a fast detection model. Specifically, a variable containing the product of the scores from a fast detection model and corresponding CNN confidences are used for ranking the final result. In the research of [74], Geng et al. applied visual attention [74] to fine-grained product classification tasks. Attention maps are employed to magnify the influences of the features, consequently to guide the CNN classifier to focus on fine discriminative details. Eventually, they compared their method with state-of-the-art approaches and obtained promising results. Based on the method of [142], George et al. performed fine-grained classification for products on a shelf in [143]. They extracted midlevel discriminative patches on product packaging and then employed SVM classifiers to differentiate visually similar product classes by analyzing the extracted patches. Their work shows the superior performance of using discriminative patches in the fine-grained product classification. In the recent study from [144], a self-attention module is proposed for capturing the most informative parts in images. The authors compared the activation response of a position with the mean value of features to locate the crucial parts of the fine-grained objects. The experimental results in [144] show that the fine-grained recognition performance has been improved in cross-domain scenarios.

#### 4.3.2. Context Awareness

Context is a statistical property of the world which provides critical cues to help us detect specific objects in retail stores [145], especially when the appearance of an object may not be sufficient for accurate categorization. Context information has been applied to improve the performance for the domain of object detection [145–147] due to its ability to provide useful information about spatial and semantic relationships between objects.

With regard to the scenario in a supermarket, products are generally placed on shelves according to certain arrangement rules, e.g., intraclass products are more likely to appear adjacent to each other on the same shelf. Consequently, context can be considered as a reference for recognising similar products on shelves, jointly with deep features. Currently, there are few works of the literature taking contextual information into account with deep learning detectors for product recognition. In [53], a novel technique to learn deep contextual and visual features for the fine-grained classification of products on shelves is introduced. Technically, authors proposed a CRF-based method [82] to learn the class embedding from a CNN concerning its neighbour's visual features. In this paper, the product recognition problem is addressed not only based on its visual appearance but also on its relative locations. This method has been evaluated on a dataset that contains product images from retail stores, and it improves the recall to 87% with 91% precision. Another two papers also obtained prominent results by considering the context. However, they did not use a deep learning-based feature descriptor. One is from [64], and it presents a context-aware hybrid classification system for fine-grained product recognition, which combines the relationships between the products on the shelf with image features extracted by SIFT methods. This method achieves an 11.4% improvement compared with the context-free method. In [148], authors proposed a computer vision pipeline that detects missing or misplaced items by using a novel graph-based consistency check method. This method regards the product recognition problem as a subgraph isomorphism between the item packaging and the ideal locations.

### 4.4. One-Shot Learning

**Figure 11:**
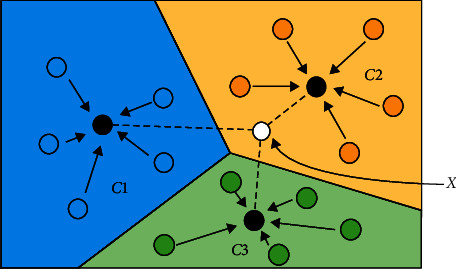
The prototype network of one-shot learning.

One-shot learning is derived from distance metric learning [149] with the purpose of learning information about object categories from one or only a few training samples/images [87]. It is of great benefit for seamlessly handling new products/packages as the only requirement is to introduce one or several images of the new item into the reference database with no or minimal retraining. The basic concept of how to classify objects with one-shot learning is shown in Figure 11. The points, *C*1, *C*2, and *C*3, are the mean centres of feature embeddings from three different categories, respectively. Based on the feature embedding of *X*, the calculation of the distance between *X* and the three points (*C*1, *C*2, and *C*3) can be conducted. Thus, *X* will be identified in the class that has the shortest distance. Additionally, one-shot learning is also a powerful method to deal with the training data shortage, with the possibility of learning much information about a category from just one or a handful of images [87]. Considering the advantages of one-shot learning, a lot of literature has combined one-shot learning with the CNN for a variety of tasks including image classification [150–153] and object detection [154, 155].

In [152], a novel metric was proposed, including colour-invariant features from intensity images with CNNs and colour components from a colour checker chart. The metric is then used by a one-shot metric learning approach to realize person identification. Vinyals et al. in [150] designed a matching network, which employs metric learning based on deep neural features. Their approach is tested on the ImageNet dataset and is able to recognize new items when introducing a few examples of a new item. Compared with the Inception classifier [41], it has increased the accuracy of one-shot classification on ImageNet from 87.6% to 93.2%. In the domain of object detection, the work in [155] combines distance metric learning with R-CNN and implements animal detection with few training examples. Video object segmentation was achieved in [154], where the authors adapted the pretrained CNN to retrieve a particular object instance, given a single annotated image, by fine-tuning on a segmentation example for the specific target object.

Two very recent papers have succeeded in addressing the specific domain of retail products to take the experience of one-shot learning combined with deep features from CNNs. In [74], a framework integrating feature-based matching and one-shot learning with a coarse-to-fine strategy is introduced. This framework performs flexibly, which allows adding new product classes without retraining existing classifiers. It has been evaluated on the GroZi-3.2k [13], GP-20 [58], and GP181 [148] datasets and attained 73.93%, 65.55%, and 85.79% for mAP, respectively. Another work from [20] proposes a pipeline which pursues product recognition through a similarity search between the deep features of reference and query images. Their pipeline just requires one training image for each product class and handles seamlessly new product packaging.

In this section, we provided a comprehensive literature review to summarize the research status of the four techniques, which are powerful tools to deal with the challenging problems of product recognition. In the next section, we introduce the public datasets and present a comparative study on the performances of deep learning methods.

## 5. Dataset Resources

As mentioned earlier, deep learning always requires plenty of annotation images for training and testing, while it is often labour-intensive to obtain labelled images in real practice. In this section, we present public dataset resources, assisting researchers in testing their methods and comparing results based on the same dataset. According to the different application scenarios, we split the resources into two categories: on-shelf and checkout.  Table 5 lists the detailed information of several available datasets, including the number of product categories, the number of instances in each image, and the number of images in the training and testing sets. The datasets are briefly introduced in the following.

### 5.1. On-Shelf Datasets

On-shelf datasets are benchmarks for testing methods proposed to recognize products on shelves, which shall benefit product management. Here, we present six available datasets.

#### 5.1.1. GroZi-120

The GroZi-120 dataset [89] consists of 120 product categories, with images representing the same products under completely different conditions, together with their text annotations. The training set includes the 676 images in vitro: such images are captured under ideal conditions. A training image just contains one single instance, enabling this dataset to suit one-shot learning. The test set has 4,973 frames annotated with ground truth in situ, which are rack images obtained from natural environments with a variety of illuminations, sizes, and poses. It also has 29 videos with a total duration of 30 minutes, including every product presented in the training set. The in situ videos are recorded using a VGA resolution MiniDV camcorder at 30 fps, and the in situ rack images are of low resolution. Samples of in vitro images and in situ rack images are shown in Figure 4.

#### 5.1.2. GroZi-3.2k

**Figure 12:**
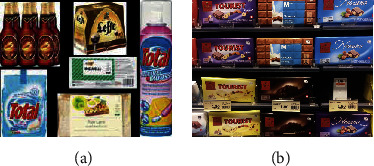
GroZi-3.2k: samples of training images (a) and testing images (b).

GroZi-3.2k [13] is a dataset containing supermarket products, which can be used in fine-grained recognition. This dataset includes 8,350 training images collected from the web, belonging to 80 broad product categories. Training images are taken in ideal conditions with a white background, and most of them only contain one single instance in each image. On the contrary, the testing set consists of 680 images captured from 5 real-life retail stores using a mobile phone, with ground truth annotations. The reason why this dataset is named as GroZi-3.2k is that all the products in the test images are from the 27 training classes of the “food” category under which 3,235 training images are included. Examples of training and testing images are shown in Figure 12.

#### 5.1.3. Freiburg Grocery Dataset

**Figure 13:**
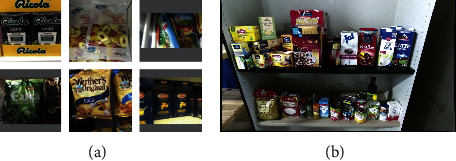
Freiburg Grocery: samples of training images (a) and testing images (b).

The Freiburg Grocery dataset [77] consists of 4,947 images of 25 grocery classes. The training images are taken at some stores, apartments, and offices in Germany using four different phone cameras. Each training image has been downscaled to a size of 256*∗*256 pixels, containing one or several instances of one category. Furthermore, an additional set includes 74 images collected in 37 cluttered scenes that can be used as a testing set. Each testing image is recorded by a Kinect v2 [156] camera at 1920*∗*1080 pixels RGB, containing several products belonging to multiple classes.  Figure 13 indicates some examples of training images and testing images from this dataset.

#### 5.1.4. Cigarette Dataset

**Figure 14:**
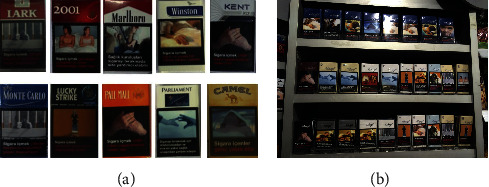
Cigarette dataset: samples of training images (a) and testing images (b).

The Cigarette dataset [157] comes with product images and shelf images from 40 retail stores, captured by four different types of cameras. The training set consists of 3,600 product images belonging to 10 cigarette classes. Each image in this set includes only one instance. The testing set is made of 354 shelf images, which have approximately 13,000 products in total. Each product in the shelf image has been annotated with bounding boxes and cigarette categories using the Image Clipper utility.  Figure 14 demonstrates the brand classes and an example of shelf images.

#### 5.1.5. Grocery Store Dataset

**Figure 15:**
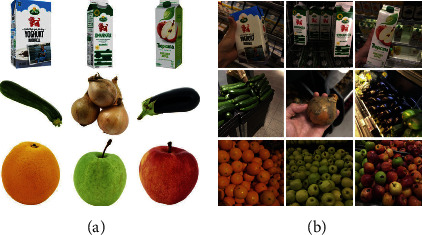
Grocery Store dataset: samples of iconic images (a) and natural images (b).

The Grocery Store dataset [158] was developed to address the natural image classification for assisting people who are visually impaired. This dataset consists of iconic images and natural images. The iconic images are downloaded from a grocery store website with product information, such as origin country, weight, and nutrient values. On the contrary, the natural images are collected images from 18 different grocery stores recorded by a 16-megapixel phone camera with different distances and angles. This set, containing 5,125 images from 81 fine-grained classes, has been split into one training set and one test set randomly to reduce the data bias. The training and test set contain 2,640 and 2,485 images, respectively, and each image contains one or several instances of one product class.  Figure 15 illustrates the examples of iconic and natural images.

#### 5.1.6. GP181 Dataset

The GP181 dataset [148] is a subset of the Grozi-3.2k dataset, with 183 and 73 images in training and testing sets, respectively. Each training image includes a single instance of one product category. Images from test sets have been annotated with item-specific bounding boxes. This dataset can be found at http://vision.disi.unibo.it/index.php?option=com_content&view=article&id=111&catid=78.

Here, we present a comparison of the product recognition performance on GroZi-120, GroZi-3.2k, and its subset in Table 6. All the methods of the listed publications are based on deep learning. The performance was calculated by using recall, precision, and accuracy. Precision measures the percentage of correct predictions over the total number of predictions, while the recall measures the percentage of correctly detected products over the total number of labelled products in the image [148]. Here are their mathematical definitions: precision = (TP/(TP + FP)), recall = (TP/(TP + FN)), and accuracy = ((TP + TN)/(TP + FN + FP + TN)), where TP, TN, FP, and FN refer to true positive, true negative, false positive, and false negative, respectively.

### 5.2. Checkout Datasets

As mentioned in the Introduction section, the scenario of recognising products for the self-checkout system is also a complex task that needs to be solved, which will benefit both retailers and customers. Since it is an emerging research area, this problem has not been extensively studied. There are two public datasets available for the checkout system.

#### 5.2.1. D2S Dataset

**Figure 16:**
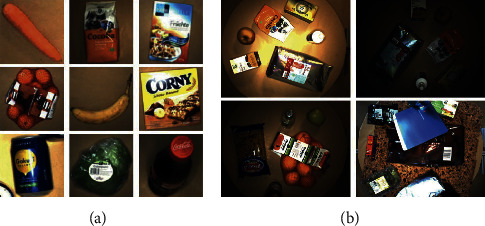
D2S dataset: samples of training images (a) and testing images (b).

The D2S dataset [80] is the first-ever benchmark to provide pixelwise annotations on the instance level, aiming to cover real-world applications of an automatic checkout, inventory, or warehouse system. It contains a total of 21,000 high-resolution images of groceries and daily products, such as fruits, vegetables, cereal packets, pasta, and bottles, from 60 categories. The images are taken in 700 different scenes under three different lightings and three additional backgrounds. The training set includes 4,380 images captured from different views, and each image involves one product of a single class. There are 3,600 and 13,020 images in the validation and test sets, respectively. Furthermore, 10,000 images in the validation and test sets are artificially synthesized that contain one to fifteen objects randomly picked from the training set. The samples of training images and test images are shown in Figure 16.

In the work of [80], the authors evaluated the performance of several state-of-the-art deep learning-based methods on the D2S dataset, including Mask R-CNN [55], FCIS [93], Faster R-CNN [50], and RetinaNet [159]. The results are summarized in Table 7. The evaluation metric is mean average precision (mAP) [57]. Specifically, mAP_50_ and mAP_75_ are calculated at the intersection-over-union (IoU) thresholds 0.50 and 0.75 over all product classes, respectively.

#### 5.2.2. RPC Dataset

**Figure 17:**
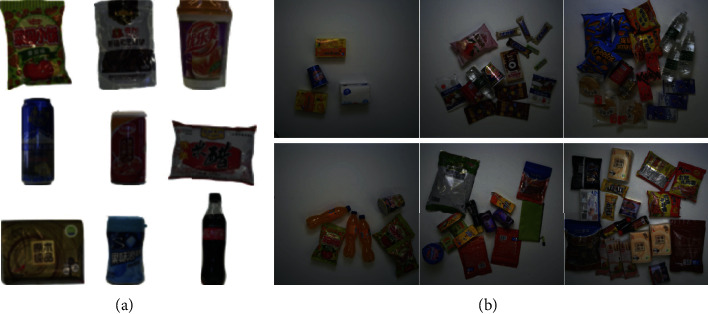
RPC dataset: samples of training images (a) and checkout images (b).

The RPC dataset [7] is developed to support research on addressing product recognition in real-world checkout scenarios. It consists of 83,739 images in total, including 53,739 single-product exemplary images for training and 30,000 checkout images for validation and testing. It has a hierarchical structure of 200 fine-grained product categories, which can be coarsely categorized as 17 metaclasses. Each training image is captured in controlled conditions with four cameras from different views. The checkout images are recorded with three clutter levels using a camera mounted on top, annotated with a bounding box and object category for each product.  Figure 17 demonstrates some examples of training images and checkout images in the RPC dataset.

In [7], feature pyramid network (FPN) [123] is adopted as the detector for recognising items on the RPC dataset, and reasonable results have been achieved in this paper. In addition, the authors also proposed an essential metric, checkout accuracy (cAcc), for the automatic checkout task in [7]. At first, CD_*i*,*k*_ is defined as the counting error for a particular category in a checkout image:

(1) $$\begin{array}{c} {\text{CD}}_{i,k}=\left|P_{i,k}-{\text{GT}}_{i,k}\right|, \end{array}$$

where *P*_*i*,*k*_ and GT_*i*,*k*_ denote the predicted count and ground-truth item number of the *k*-th class in the *i*-th image, respectively. Then, the calculation of the error over all *K* product classes in the *i*-th image is defined as

(2) $$\begin{array}{c} {\text{CD}}_{i}=\sum_{k=1}^{K}{\text{CD}}_{i,k}. \end{array}$$

Given *N* images from the RPC dataset, cAcc measures the mean accuracy rate of the correct predictions. Its mathematical definition is

(3) $$\begin{array}{c} \text{cAcc}=\frac{\sum_{i=1}^{N}\delta \left({\text{CD}}_{i},0\right)}{N}, \end{array}$$

where *δ*(*·*) = 1 if CD_*i*_ = 0; otherwise, it equals 0. The value of cAcc ranges from 0 to 1.

Afterwards, based on the work of Wei et al. [7], data priming network (DPNet) was developed to select reliable samples to promote the training process in [78]. Consequently, the performance of product recognition has been significantly boosted with DPNet. The comparative results of [7, 78] are listed in Table 8, where mmAP is the mean value over all 10 IoU thresholds (i.e., ranging from 0 : 50 to 0 : 95 with the uniform step size 0 : 05) of all product classes [7].

## 6. Research Directions and Conclusion

To the best of our knowledge, this paper is the first comprehensive literature review on deep learning approaches for retail product recognition. Based on the thorough investigation into the research of retail product recognition with deep learning, this section outlines several promising research directions for the future. Finally, we present a conclusion for the whole article.

### 6.1. Research Directions

#### 6.1.1. Generating Product Images with Deep Neural Networks

In the previous introduction of dataset resources, the largest publicly available dataset only contained 200 product categories. Nevertheless, the number of different items to be recognized in a medium-sized grocery store can be approximately several thousands, far exceeding the category quantity of the existing datasets. Considering the appearances of existing products frequently change over time, it is impossible to build a man-made dataset that includes the majority of daily products. Some works [7, 71, 78] have demonstrated the advantages of generative adversarial networks (GANs) for generating images that look realistic. Moreover, significant work in [102] has filled the gap between CNNs and GANs by proposing the deep convolutional generative adversarial networks (DCGANs) that can create high-quality generated images. In this case, it is feasible to generate images with deep neural networks to enlarge the training dataset for retail product recognition. So, developing image generators with deep neural networks to simulate real-world scenes shall be a future research direction.

#### 6.1.2. Graph Neural Networks with Deep Learning for Planogram Compliance Check

Graph neural networks (GNNs) [160] are a powerful tool for non-Euclidean data, which can represent the relationships between objects [161, 162]. Currently, GNNs have achieved great success on recommendation systems [163, 164], molecule identification [165], and paper citation analysis [166]. For an image that contains multiple objects, each object can be considered as a node, and GNNs have the ability to learn the location relationship between every two nodes. With regard to the scenarios in supermarkets, products are generally placed on shelves according to certain arrangement rules. In this case, GNNs can be used with deep learning to learn the position relationships between different products, and then they are assisted by identifying missing or misplaced items for planogram compliance. In [148], authors attempted to apply GNNs for consistency checks and achieved a remarkable result. Specifically, there are two relationship representations. One is “observed planogram” generated from GNNs, and another one is “reference planogram,” the true representation. By comparing the observed planogram and reference planogram, they obtained the result of the consistency check that helps to correct the false detection and missing detection.

#### 6.1.3. Cross-Domain Retail Product Recognition with Transfer Learning

In object detection algorithms, a significant assumption is that the learning and test data are derived from the same feature space and the same distribution [167], i.e., most object detectors require retraining with new data from random initialization when the distribution changes. In the real world, many different retail stores and supermarkets are selling diversified products. Moreover, the internal environment between different shops can be varied. One model trained by data from a specific shop is unable to be applied with a newly built store, which arises the concept of cross-domain recognition. Cross-domain recognition is usually based on transfer learning [168] that assists the target domain in learning by using knowledge transferred from other domains. Transfer learning is capable of solving new problems easily by applying knowledge obtained previously. For a new task, researchers normally use the pretrained detector either as an initialization or a fixed feature extractor and then fine-tune the weights of some layers in the network to realize cross-domain detection. Ordinarily, the majority of approaches in established papers employs models pretrained with ImageNet to implement product recognition [20, 74]. However, how to make a model adaptable in various shops still needs attention.

#### 6.1.4. Joint Feature Learning from Text Information on Product Packaging

Intraclass product classification is a challenge since it is visually similar. Sometimes, we human beings recognize similar products by reading the text on packaging when we are facing a lot of intraclass items. Thus, the text information on product packaging can be considered as a factor for classifying fine-grained products. Currently, joint feature learning (JFL) methods have shown their effectiveness in improving the face recognition performance by stacking features extracted from different face regions [169]. For this reason, it is possible for the idea of JFL to be introduced to the field of retail product recognition, i.e., learning the product image features and package text features jointly to enhance the recognition performance. In [143], researchers tried to automatically recognize the text on each product packaging. Unfortunately, the extracted text information in this paper is just used to search for products for users.

#### 6.1.5. Incremental Learning with the CNN for Flexible Product Recognition

Deep learning methods always suffer from “catastrophic forgetting,” especially for convolutional neural networks, i.e., they are incapable of recognising some previously learned objects when adjusted to a new task [65]. Incremental learning is a powerful method that can deal with new data without retraining the whole model. Additionally, it enables deep neural networks to have a long-term memory. Shmelkov et al. and Guan et al. [65, 170] implemented incremental learning of object detection by proposing two detection networks. One is an existing network that has already been trained, and the other one will be trained for detecting new classes. In [171], authors attempted to combine incremental learning with CNNs and compared various incremental teaching approaches for CNN-based architectures. Therefore, incremental learning will be helpful to make the recognition system flexible with no or minimal retraining whenever a fresh item is launched.

#### 6.1.6. The Regression-Based Object Detection Methods for Retail Product Recognition

If we want to apply product recognition in the industry area, it requires real-time availability. Consumers would like to check out immediately, and retailers shall receive real-time feedback when something is missing from the shelves. As we all know, deep learning is computationally expensive. A large number of deep learning algorithms need to use GPUs to run image processing. As mentioned in Section 2, there are two categories of the object detection methods: region proposal-based and regression-based [47]. The regression-based methods can reduce the time expense by regressing the objects' locations and categories directly from image pixels [54]. Ordinarily, the regression-based methods perform better for real-time detection tasks than the methods based on region proposals. However, although the work in [51] achieves detection of general objects at a high rate of speed, it suffers from accuracy reduction. Therefore, how to improve the detection accuracy with the regression-based approach for retail product recognition is worth more research.

### 6.2. Conclusion

This paper addresses the broad area of product recognition technologies. Product recognition will become increasingly important in a world where cost margins are becoming increasingly tight, and customers have increasing pressures on their available time. By summarising the literature in the field, we make research in this area more accessible to new researchers, allowing for the field to progress. It is very important that this field addresses these four challenging problems: (1) large-scale classification; (2) data limitations; (3) intraclass variation; and (4) flexibility. We have identified several areas for further research: (1) generating data with deep neural networks; (2) graph neural networks with deep learning; (3) cross-domain recognition with transfer learning; (4) joint feature learning from text information on packaging; (5) incremental learning with the CNN; and (6) the regression-based object detection methods for retail product recognition.

In this article, we have presented an extensive review of recent research on deep learning-based retail product recognition, with more than one hundred references. We propose four challenging problems and provide corresponding techniques to those challenges. We have also briefly described the publicly available datasets and listed their detailed information, respectively.

Overall, this paper provides a clear overview of the current research status in this field and that it encourages new researchers to join this field and complete extensive research in this area.

## Figures and Tables

**Table 1:** Image labelling tools.

Categories	Tools	Environment
Bounding box	labelImg (https://github.com/tzutalin/labelImg)	Python
	bbox-label-tool (https://github.com/puzzledqs/BBox-Label-Tool)	Python
	LabelBoundingBox (https://github.com/hjptriplebee/LabelBoundingBox)	Python
	YOLO_*m*_*ark* (https://github.com/AlexeyAB/Yolo_mark)	Python
	CVAT (https://github.com/opencv/cvat)	Python
	RectLabel (https://rectlabel.com/)	Mac OS
	VoTT (https://github.com/microsoft/VoTT)	Java/Python

Mask	labelme (https://github.com/wkentaro/labelme)	Python
	Labelbox (https://github.com/Labelbox/Labelbox)	Java/Python

**Table 2:** CNN-based feature descriptors and relevant approaches where these descriptors are employed.

Feature descriptors	Approaches
Inception [70]	[71, 72]
GoogLeNet [41]	[67]
AlexNet [15]	[21, 53, 58, 67, 73]
VGG [42]	[20, 21, 71, 74–76]
CaffeNet [35]	[10, 67, 73, 77]
ResNet [43]	[22, 68, 71, 74, 78–80]

**Table 3:** Related works for data limitation in the field of retail product recognition.

Technique	Categories	Existing works
Data augmentation	Common synthesis	[22, 76, 80]
		[21, 79, 89]
	Generative	[7, 71, 78]

**Table 4:** Summary of models based on the structures of VAE and GAN for image synthesis.

Synthesis type	VAE	GANs
Image generation	VAE [96]	GAN [97]
	cVAE [99]	CGAN [100]
	Attribute2Image [101]	DCGAN [102]
	Multistage VAE [103]	InfoGAN [104]

Image translation	—	Pix2Pix [105]
		CycleGAN [106]
		DualGAN [107]
		DiscoGAN [108]
		StarGAN [109]
		VAE-GAN [110]

**Table 5:** Detailed information of several public datasets.

Scenario	Dataset	#product categories	Training set		Test set	
			#instances per image	#number of images	#instances per image	#number of images
On-shelf	GroZi-120 dataset (http://grozi.calit2.net/grozi.html)	120	Multiple	676	Multiple	4,973
	GroZi-3.2k dataset (https://sites.google.com/view/mariangeorge/datasets)	27/80	Single	8,350	Multiple	3,235
	Freiburg Grocery dataset (https://github.com/PhilJd/freiburg_groceries_dataset)	25	Multiple (one class)	4,947	Multiple	74
	Cigarette dataset (https://github.com/gulvarol/grocerydataset)	10	Single	3,600	Multiple	354
	Grocery Store dataset (https://github.com/marcusklasson/GroceryStoreDataset)	81	Multiple (one class)	2,640	Multiple (one class)	2,458

Checkout	D2S dataset (https://www.mvtec.com/company/research/datasets/mvtec-d2s/)	60	Single	4,380	Multiple	16,620
	RPC dataset (https://rpc-dataset.github.io/)	200/17	Single	53,739	Multiple	30,000

**Table 6:** Recognition performance comparison of approaches based on deep learning on benchmark datasets.

Publications	GroZi-120 [89]			GroZi-3.2k [13]		
	Precision (%)	Recall (%)	#product categories	Precision (%)	Recall (%)	#product categories
[58]	45.20	52.70	120	73.10	73.60	20
[20]	—	—	—	73.50	82.68	181
[148]	—	—	—	90.47	90.26	181
[71]	—	—	—	Accuracy: 85.30		181
[74]	49.05	29.37	120	65.83	45.52	857
				92.19	87.89	181
[21]	49.80	—	120	52.16	—	27

**Table 7:** Product detection benchmark results on the test set of the D2S dataset.

Approaches	mAP (%)	mAP_50_ (%)	mAP_75_ (%)
Mask R-CNN [55]	78.3	89.8	84.9
FCIS [93]	68.3	88.5	80.9
Faster R-CNN [50]	78.0	90.3	84.8
RetinaNet [159]	80.1	89.6	84.5

**Table 8:** Comparative results on the RPC dataset.

Publications	cAcc (%)	mAP_50_ (%)	mmAP (%)
[7]	56.68	96.57	73.83
[78]	80.51	97.91	77.04
